# Pronounced Diurnal Pattern of Salivary C-Reactive Protein (CRP) With Modest Associations to Circulating CRP Levels

**DOI:** 10.3389/fimmu.2020.607166

**Published:** 2021-01-08

**Authors:** Jonas Wetterö, Sarah von Löhneysen, Flordelyn Cobar, Margareta Kristenson, Peter Garvin, Christopher Sjöwall

**Affiliations:** ^1^Division of Inflammation and Infection, Department of Biomedical and Clinical Sciences, Linköping University, Linköping, Sweden; ^2^Faculty of Mathematics and Computer Science, University of Leipzig, Leipzig, Germany; ^3^Division of Rheumatology, Department of Medicine Solna, Karolinska Institutet and Rheumatology, Karolinska University Hospital, Stockholm, Sweden; ^4^Division of Society and Health, Department of Health, Medicine and Caring Sciences, Linköping University, Linköping, Sweden; ^5^Research and Development Unit in Region Östergötland, Department of Health, Medicine and Caring Sciences, Linköping University, Linköping, Sweden

**Keywords:** saliva, C-reactive protein, pentraxins, inflammation, interleukin 6, biomarker

## Abstract

C-reactive protein (CRP), a humoral component of the innate immune system with important functions in host-defense, is extensively used as a sensitive biomarker of systemic inflammation. During inflammation, hepatocyte-derived CRP rises dramatically in the blood due to increased interleukin-6 (IL-6) levels. Reliable detection of CRP in saliva, instead of blood, would offer advantages regarding sampling procedure and availability but using saliva as a diagnostic body fluid comes with challenges. The aims of this study were to evaluate associations between salivary CRP, total protein levels in saliva and serum CRP. Furthermore, we examined associations with plasma IL-6, body mass index (BMI), tobacco smoking and age. Salivary CRP was investigated by ELISA in 107 middle-aged participants from the general population. We employed spectrophotometric determination of total protein levels. Correlation analyses were used for associations of salivary CRP with serum CRP (turbidimetry), plasma IL-6 (Luminex^®^), BMI and smoking habits. Salivary median CRP was 68% higher (*p*=0.009), and total protein levels were 167% higher (*p*<0.0001), in morning compared to evening saliva. The correlation coefficients between serum and salivary CRP were low to moderate, but stronger for evening than morning saliva. Plasma IL-6 correlated significantly with serum CRP (*r_s_*=0.41, *p*<0.01), but not with morning or evening salivary CRP. Non-smokers showed 103% higher salivary CRP levels (*p*=0.015), whereas serum CRP was independent of smoking status. As opposed to CRP in serum, salivary CRP was not associated with BMI. Salivary CRP was 90% higher among the age interval 60–69 years compared to subjects aged 45–59 (*p*=0.02) while serum CRP levels did not differ between the age groups. In conclusion, CRP in saliva did not straightforwardly reflect serum concentrations. This raises questions regarding adequate reflection of biological events. The pronounced diurnal salivary CRP pattern accentuates the importance of standardizing the time-point of sampling.

## Introduction

The classical acute phase reactant C-reactive protein (CRP), originally described by Tillett & Francis in 1930, belongs to the pentraxin family and consists of five identical 23-kDa globular subunits (1, 2). CRP plays multiple important roles in innate immunity and host-defense. It binds to specific ligands like phosphorylcholine and activates the classical complement pathway through complement protein (C) 1q binding (3, 4).

Similarly to immunoglobulin G (IgG), CRP also has a binding site for Fc-γ receptors of phagocytic cells. However, in contrast to antibodies, ligand binding is less specific and does not require affinity maturation. Furthermore, the capacity of CRP to recognize nuclear components on the surface of apoptotic cells, and thereby to facilitate their clearance, could potentially act as a protector against autoimmunity (5). The pentameric structure of CRP is dependent upon the presence of calcium, although it can also be disrupted irreversibly into monomers under denaturing conditions, such as an acidic local microenvironment. Monomeric/modified CRP can be deposited in tissues, but the biological relevance of this remains unclear and warrants further investigation (5–8).

As a marker of acute inflammation, CRP has a short half-life of about 19 h in the circulation and declines rapidly after resolution of the acute phase response and it is widely used clinically to monitor infections, particularly those caused by bacteria, and inflammatory diseases, both acute infections and chronic conditions (5). In systemic lupus erythematosus, however, the circulating levels of CRP are generally low, despite inflammatory activity. Although some investigators have reported extrahepatic CRP production, human CRP is mainly synthesized by primary hepatocytes after stimulation through its main inducer interleukin (IL) 6, but impeded by interferon-α (9, 10). CRP has been thoroughly studied as a biomarker of future cardiovascular events (11, 12). Whether or not CRP plays a causal role in the development of atherosclerosis is debated (13).

The interest of mucosal immunity in general and the use of human *salivary* CRP as a diagnostic body fluid is increasing (14). Saliva contains a unique mixture of proteins, nucleic acids, electrolytes and hormones derived from systemic as well as local sources (15). Advantages of using saliva as a diagnostic tool are that the collection is non-invasive, relatively easy and rapid, safe, stress- and pain-free and cheap because sampling is possible without professionals (15–17). Yet there are also challenges in handling saliva. Biomarkers are often less concentrated in saliva than in serum, and therefore harder to detect by traditional analytic approaches, and their concentrations can be influenced by circadian cycles or especially sampling/processing methods (15). In addition, saliva collection can be performed either by a “passive” approach, meaning that the saliva is gathered in the mouth and then transferred into a sampling tube, or by an “active” mechanically stimulated approach. The latter stimulation of salivary flow is achieved by chewing on a solid object. It is also possible to use cotton swabs, which absorb the saliva, and then release saliva by centrifugation. Unstimulated saliva originates mainly from the submandibular gland whereas parotid saliva production increases intensively with stimulation (18). All of these sources of variation must be considered to evaluate the usefulness of saliva as a diagnostic fluid. Regarding CRP, an essential question is if salivary levels reflect the same biological events as the serum levels do. Different studies investigated the association between salivary and serum concentrations with inconsistent results (19–22). Some indications of a diurnal rhythm of salivary CRP with elevated levels in the morning have been reported (20, 23).

In the present study, we asked whether CRP levels vary similarly to the total protein levels in saliva, and if any substantial diurnal variation occurs. The general aim was thus to evaluate associations between salivary CRP, total protein levels in saliva and serum CRP in collected samples from middle-aged individuals of the general population. In addition, we examined associations between salivary CRP and body mass index (BMI), tobacco smoking and age.

## Materials and Methods

### Participants

In the present study, samples were obtained from 107 participants (61 men, 46 women; median age of 57, range 45–69 years) randomly selected from the *Life conditions, Stress and Health Study* (LSH) cohort at Linköping University, aimed at investigating the pathways that link psychosocial factors to cardiovascular diseases (24). The LSH cohort is almost ten times larger (*n*=1007) and includes individuals who were randomly chosen from the general population in the Region of Östergötland, Sweden. Participants in the LSH cohort provided saliva and blood samples and answered questionnaires regarding their socioeconomic, psychological and health status. Exclusion criteria were self-reported severe disease that hindered the possibility to participate, *e.g.* terminal cancer, severe dementia and psychiatric disorders. Participants with symptoms of infection were instructed to return for sampling after recovery (24).

### Collection of Saliva

The saliva was sampled at home at three time-points over three consecutive days (*d*_1_, *d*_2_, *d*_3_) using Salivette^®^ cotton swabs (Sarstedt AG & Co., Nümbrecht, Germany). For saliva collection, the swab was placed in the mouth and only chewed on if the salivation was low and participants felt discomfort or mouth dryness. The participants were instructed to take samples immediately after awakening (*t*_1_), 30 min after awakening (*t*_2_), and just before going to bed (*t*_3_) following their normal sleeping habits. The reported time for *t*_1_ was 6:30 (mean and median), range 04:23 to 11:00, for *d*_1_, *d*_2_ and *d*_3_ (standard deviation 57 min). For >75% of the study participants, samples were collected between 5:30 and 7:30. The reported time for *t*_3_ was 22:15 (mean and median), range 19:00 to 0:35 (standard deviation 65 min). For >75% of the study participants, samples were collected between 21:15 and 23:15. Participants were also told to avoid physical exercise, food intake and smoking 1 h prior to sampling. After collection, the samples were stored in a refrigerator until centrifugation. Thereafter, samples were frozen at –70°C until analysis (25). To standardize for any potential differences in viscosity of the saliva, the samples used herein were centrifuged at 1500*g* for 15 min after thawing, and aliquots were taken from the supernatants for upcoming analyses.

### Serum C-Reactive Protein and Plasma IL-6

All blood samples were obtained at primary health care centers between 6.30 and 9.00 AM (typically between 7:30 and 8:00 AM) in a fasting state of the first day of the week following the saliva-sampling period (*nota bene* salivary CRP was measured in the samples from *d*_3_). Aliquots of sera were stored at –70°C until analysis (24, 25). CRP levels were detected in serum utilizing a highly sensitive latex-enhanced turbidimetric immunoassay (Roche Diagnostics GmbH, Vienna, Austria) with a lower detection limit of 0.03 mg/L, the coefficient of variance (CV) was 1.7% and the detection rate of 100% (26).

Levels of IL-6 were determined in ethylenediaminetetraacetic acid (EDTA)-plasma with an ultra-sensitive bead kit technology (Invitrogen Co., Carlsbad, CA, USA) on a Luminex^®^ 100TM system (Austin, TX, USA). The lower detection limits were set at 1.68 pg/ml for IL-6. The proportion of samples with levels above this limit was 40% for IL-6. The CV was 7.0% for IL-6.

### Body Mass Index and Tobacco Smoking

The participants’ BMI were calculated based on weight in kilograms divided by the square of their height in meters (median BMI 23.3, range 18.7–39.5). The division of tobacco smokers (17.3%) and non-smokers (82.7%) was based on self-reported data from the questionnaires. Participants smoking ≥1 cigarette per day were considered as “current smoker”. All participants reporting ‘never smoked’ or “quit smoking” were categorized as “non-smokers”.

### Salivary C-Reactive Protein Assay

CRP in saliva was measured in morning (*t*_1_) and evening (*t*_3_) samples of the third sampling day (*d*_3_) using Salimetrics’ salivary CRP ELISA kit (State College, PA, USA) according to the manufacturer’s instruction. The optical density was read on a Sunrise™ microplate reader (Tecan, Männedorf, Switzerland) at 450 nm (reference wavelength 630 nm) and measurement data were processed using Magellan^™^ software V.7.1 (Tecan). All samples were analyzed in duplicates as a pilot study in samples from healthy donors that had been spiked with CRP isolated from human plasma (Sigma-Aldrich Co., St. Louis, MO, USA) showed this to be sufficiently reliable when we tested for intra-assay precision. Further, the impact of thawing-freezing cycles of the samples was investigated and CRP levels were observed to be largely stable for at least four times of thawing. For the measurements, the saliva samples were diluted 1:10 in dilution buffer (supplied with the kit). If CRP levels above the higher detection limit (=3 ng/ml) occurred, higher dilution factors were assessed. The within assay CV was 5.4% and the between assay CV 11.4%.

### Total Protein Measurements

Total protein was quantified in morning (*t*_1_) and evening (*t*_3_) saliva of two sampling days (*d*_1_ and *d*_3_), giving four values for each participant. Concentrations were obtained by measuring the absorbance at 280 nm with a Nanodrop^®^ ND-1000 spectrophotometer (Thermo Fisher Scientific, Wilmington, DE, USA) using the Nanodrop^®^ ND-1000 software V.3.8.1. A general reference setting was utilized based on the assumption that a 0.1% (1 mg/ml) protein solution produces an absorbance of 1.0 AU (Absorbance Unit) at 280 nm. Amino acids with aromatic rings are the primary reason for the absorbance peak at 280 nm. All samples were analyzed undiluted in duplicates (CV=1.2%).

### Statistics

Analyses were performed using SPSS^®^ v.20 (IBM Co., Armonk, NY, USA) and figures made in GraphPad Prism 5.03 (GraphPad Software, La Jolla, CA, USA) or in R statistical software, version 3.5.1 (Vienna, Austria). P-values <0.05 were considered statistically significant. IL-6 and salivary CRP levels below the lower detection limit were given half the limit’s value. To investigate the influence of sex and age on CRP, Mann-Whitney *U* ranking analysis was employed. Regarding age, participants <60 years of age (*n*=65) were compared to those ≥60 years (*n*=42). Correlations between salivary morning/evening CRP, total protein levels, serum CRP, BMI and plasma IL-6 were investigated by Spearman (*r_s_*). Bland–Altman graphs were used to illustrate and analyze the level of agreement between CRP in saliva and serum. Diurnal variations of CRP, total protein and CRP-percentages of total protein, respectively, were investigated by Wilcoxon. To evaluate if the differences between morning and evening values were the same for high and low CRP levels and ratios, the participants were grouped into two classes by morning salivary CRP (≤1.3 ng/ml; >1.3 ng/ml). To examine if high CRP levels (>median) coincide with high levels of BMI and IL-6 and if smokers had elevated CRP levels, we used Mann-Whitney *U* test. For this analysis, the daily mean salivary CRP concentration was employed. To evaluate the performance of salivary CRP to identify (biomarker-defined) “systemic inflammation”, the 90^th^ percentile of serum CRP values among the 107 participants was used to define a cut-off. Sensitivity (proportion of samples correctly identified with systemic inflammation), specificity (proportion of samples correctly identified without systemic inflammation) and accuracy (proportion of correctly classified samples) were calculated, including 95% confidence intervals (CI) using the Wilson score method.

## Results

### C-Reactive Protein Concentrations in Saliva and Serum

**Figure 1:**
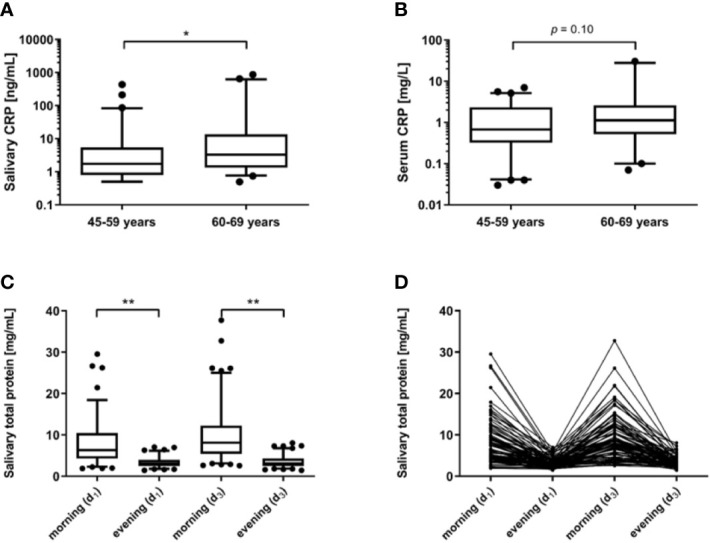
C-reactive protein (CRP) in **(A)** saliva samples (*n*=102, shown are mean values of measurements in morning and evening saliva), and **(B)** sera (*n*=96). Participants were grouped into two age classes (45–59 years and 60–69 years, respectively) and compared using Mann-Whitney *U* test. Box plots reflect the interquartile range (IQR) with the median shown as a line and the 5^th^–95^th^ percentiles as bars (* *p*<0.05). Spectrophotometric measurement of salivary total protein levels in morning and evening samples of the first (*d*_1_) and third day (*d*_3_) of sampling. **(C)** Differences in morning and evening levels of the same day were identified by Wilcoxon tests. Box plots reflect the IQR with the median shown as a line and the 5^th^–95^th^ percentiles as bars (** *p*<0.01). **(D)** The four related total protein levels shown for each participant.

Salivary morning CRP levels ranged from 0.97 to 1674.20 ng/ml with median and interquartile range (IQR) 2.44 (0.50–10.80) ng/ml. Salivary evening CRP ranged <0.97–223.9, median (IQR) 1.45 (1.05–2.72) ng/ml (**Table 1**). The detectability rate for 1:10 diluted saliva samples was 77%. Twelve outliers of morning and eight of evening CRP samples were identified exceeding four times the respective IQR. Five participants where outliers regarding both morning, evening and serum CRP. The high levels of CRP in saliva were not due to visible blood contamination in these individuals. To avoid confounding contributions from blood in saliva, visibly blood contaminated samples were excluded from further statistical analyses, although their CRP values were within the normal range (0.5–9.42 ng/ml). Serum CRP concentrations ranged from 0.03 to 30.43 mg/L with median IQR 0.90 (0.34–2.43 mg/L) (**Table 1**). No statistically significant differences were seen between men and women for any of the measures; salivary morning, evening or serum CRP concentrations. Participants aged 60–69 years had significantly higher salivary CRP levels than those aged 45–59 years (*p*=0.02, *n*=102; IQR=3.28 ng/ml (1.33–13.49) and 1.73 ng/ml (0.79–5.46), **Figure 1A**), but no significant difference in serum CRP was observed (**Figure 1B**) and neither between men and women.

**Table 1:** Descriptive statistics for C-reactive protein (CRP) levels in saliva and serum (levels below the detection limit were given the value 0.5 ng/mL; morning, *n*=27, and evening, *n*=19).

	Salivary CRP; morning [ng/ml]	Salivary CRP; evening [ng/ml]	Serum CRP [mg/L]
Range	<0.97–1674.2	<0.97–223.9	0.03–30.4
Mean (SD)	57.6 (222.75)	6.0 (24.07)	2.4 (4.68)
Median (IQR)	2.44 (0.50–10.80)	1.45 (1.05–2.72)	0.90 (0.34–2.43)
*n*	102	100	99

SD, standard deviation; IQR, interquartile range (25^th^ to 75^th^ percentiles).

### Diurnal Variation of Salivary C-Reactive Protein and Total Protein

**Figure 2:**
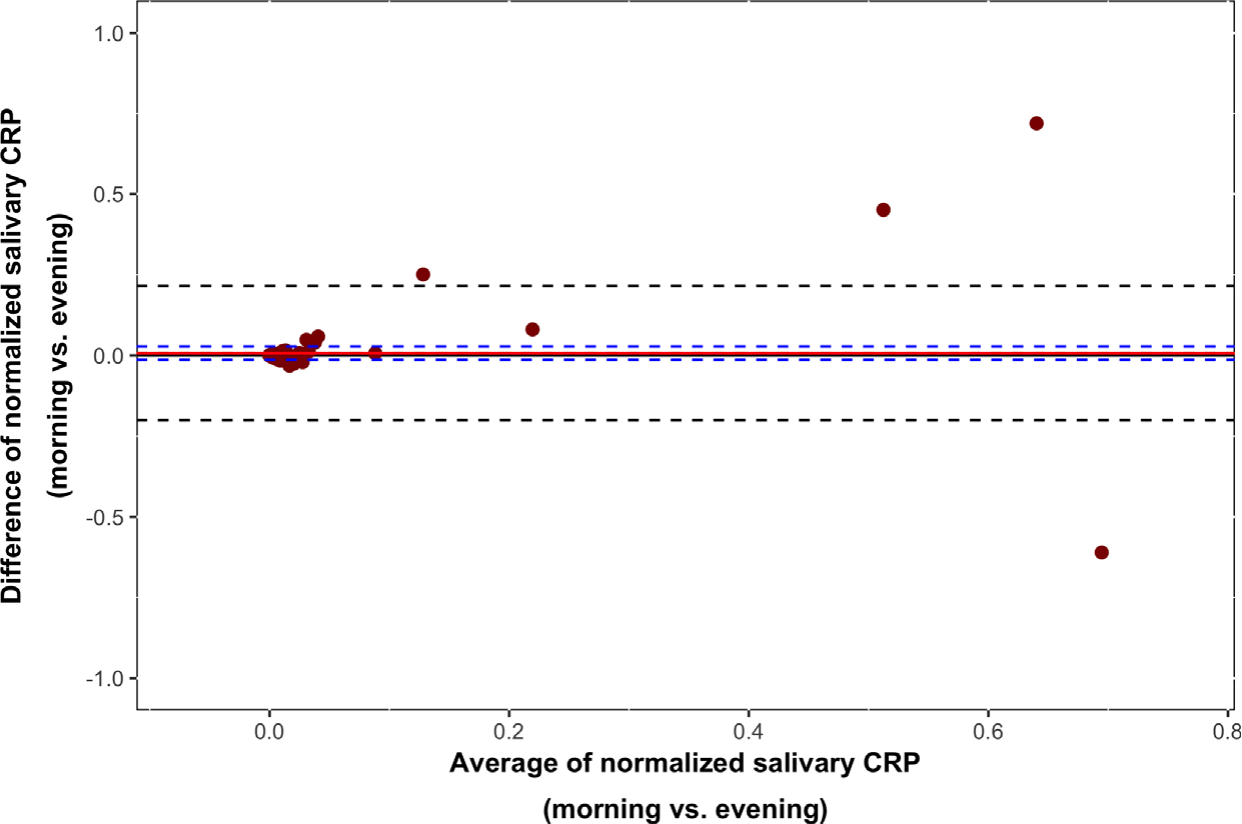
A Bland–Altman graph illustrating the level of agreement between salivary morning and evening C-reactive protein (CRP) levels based on analysis of 100 participants. The level of agreement suggested interchangeability (*p*=0.48).

**Figure 3:**
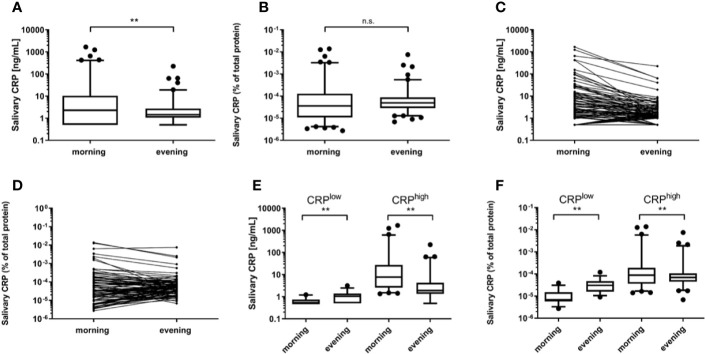
C-reactive protein (CRP) levels in morning and evening saliva samples. Displayed are absolute CRP levels **(A)** and CRP levels relative to the total protein concentrations **(B)**. Related morning and evening absolute CRP concentrations **(C)** and percentages **(D)** are shown for each participant. Morning CRP levels were classified as low (≤1.3 ng/ml) and high (>1.3 ng/ml) concentrations, respectively, and each group was compared to the related evening values. Given are the results for absolute CRP **(E)** and CRP percentages of total protein **(F)**. Differences in morning and evening levels of the same day were identified by Wilcoxon tests. Box plots reflect the IQR with the median shown as a line and the 5^th^–95^th^ percentiles as bars. Significant differences are highlighted by ** *p*<0.01 (n.s., not significant).

Total protein levels ranged from 1.46 to 37.76 mg/ml with a mean [standard deviation (SD)] of 6.92 (5.17) mg/ml. Levels at all four sampling occasions (*d*_1_: morning and evening, *d*_3_: morning and evening) correlated significantly (*r_s_*=0.37–0.78; *p*<0.01). Strongest correlations were found for the two morning (*r_s_*=0.61, *p*<0.001) and the two evening measurements (*r_s_*=0.78, *p*<0.001) (**Table 2**). A significant decrease of the total protein levels toward the evening was observed for both days of sampling (*d*_1_: *p*<0.001, *n*=87; *d*_3_: *p*<0.001, *n*=101) (**Figures 1C, D**). IQRs were 6.20 (4.20–9.55) mg/ml (morning) and 2.97 (2.42–3.89) mg/ml (evening) for *d*_1_ and 8.10 (5.42–12.25) mg/ml (morning) and 3.10 (2.53–9.55) mg/ml (evening) for *d*_3_. An additional test performed on morning and evening samples from 5 individuals (laboratory personnel) confirmed these findings (data not shown).The correlation between salivary morning and evening CRP levels was strong (*r_s_*=0.64, *p*<0.001) and, as illustrated in **Figure 2**, the Bland–Altman graph revealed a fair level of agreement (*p*=0.48). Higher CRP levels in mornings compared to evenings were found (*p*=0.009, *n*=100) (**Figures 3A, C**), but this difference disappeared after adjustment for total protein (*p*=0.59, *n*=100) (**Figures 3B, D**).

**Table 2:** Spearman (*r_s_*) correlations between total protein measures originated from morning and evening saliva collected at day 1 (*d*_1_) and day 3 (*d*_3_) of the sampling period.

	Salivary total protein; evening (*d*_1_)	Salivary total protein; morning (*d*_3_)	Salivary total protein; evening (*d*_3_)
Salivary total protein; morning (*d*_1_)	*r_s_* = 0.45**	*r_s_* = 0.61***	*r_s_* = 0.39**
	*n* = 93	*n* = 94	*n* = 94
Salivary total protein; evening (*d*_1_)		*r_s_* = 0.38**	*r_s_* = 0.78***
		*n* = 93	*n* = 93
Salivary total protein; morning (*d*_3_)			*r_s_* = 0.37**
			*n* = 101

**p < 0.01.

***p < 0.001.

To specifically investigate the diurnal patterns, the morning samples were stratified into two groups: low (≤1.3 ng/ml) and high (>1.3 ng/ml) CRP levels. In the CRP^high^ subgroup, morning levels were significantly higher regardless when absolute CRP or CRP/total protein percentage were compared with the corresponding evening samples (both *p*<0.01, *n*=63). IQRs were 7.67 (2.62–28.19) ng/ml (morning) and 1.90 (1.34–4.27) ng/ml (evening) or 8.89×10^-5^ (3.56×10^-5^–19.80×10^-5^) % (morning) and 6.95×10^-5^ (4.49×10^-5^–10.64×10^-5^) % (evening). On the contrary, the CRP^low^ subgroup showed significantly lower absolute CRP levels and CRP percentages in the morning, than in the evening (*p*<0.01, *n*=37) (**Figures 3E, F**). The IQRs were 0.50 (0.50–0.74) ng/ml (morning) and 1.05 (0.50–1.37) ng/ml (evening) or 0.68×10^-5^ (0.57×10^-5^–1.50×10^-5^) % (morning) and 3.07×10^-5^ (1.60×10^-5^–4.74×10^-5^) % (evening).

To further evaluate the impact of time in relation to saliva collection, a *post hoc* analysis was performed. We assessed any potential correlation between actual time-point of sampling and CRP in morning as well as evening saliva. None of these analyses reached statistical significance.

### Associations Between Salivary C-Reactive Protein With Serum C-Reactive Protein

**Figure 4:**
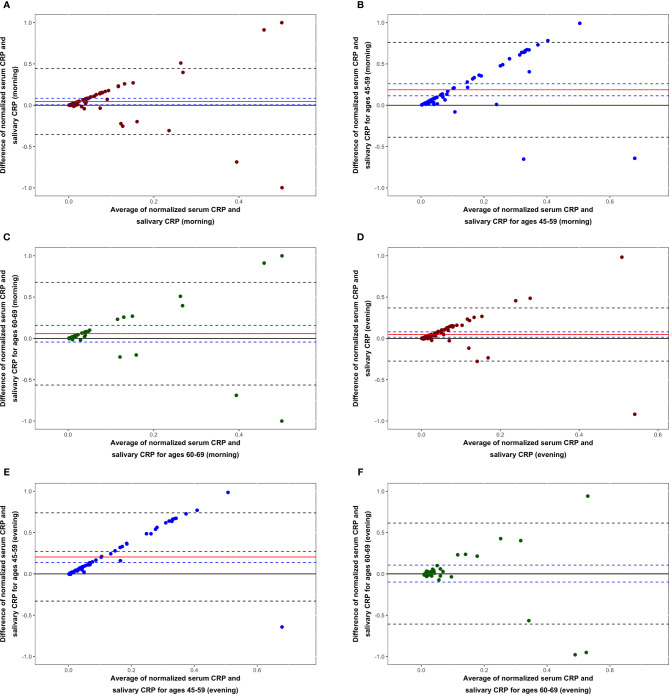
Bland–Altman graphs showing the level of agreement between serum C-reactive protein (CRP) and CRP in morning saliva for all participants **(A)**, those aged 45–59 **(B)** and those aged 60–69 years **(C)**. Bland–Altman graphs reflecting the level of agreement between serum CRP and CRP in evening saliva for all participants **(D)**, those aged 45–59 **(E)** and those aged 60–69 years **(F)**. Data are based on analysis of 99 morning and 97 evening samples. Interchangeability was only suggested among participants aged 60–69 years for morning (*p*=0.26) as well as evening (*p*=0.94) saliva.

Based on mean values of morning and evening saliva divided by serum CRP, the achieved saliva-to-serum concentration ratio reached 1:1084. We found low correlation between salivary evening CRP and serum CRP (*r_s_*=0.27, *p*<0.01), but the level of agreement was poor (*p*=0.0045). As opposed to evening salivary CRP, morning CRP levels were not significantly correlated with serum CRP and the level of agreement was mediocre (**Figure 4A**). In the 60–69 year age interval, the level of agreement was best and both morning (*p*=0.26) and evening (*p*=0.94) salivary CRP could be used interchangeably with serum CRP (**Figures 4B–F**).

We further explored the strength of correlation below and above a clinically relevant serum CRP cut-off at ≥3 mg/L (11). Salivary evening CRP showed stronger correlation with serum CRP for high serum values (*r_s_*=0.54, *p*<0.05) than for low (*r_s_*=0.28, *p*<0.01), whereas this classification did not affect correlations between salivary morning CRP and serum CRP (**Table 3**). When high salivary CRP values (>10 ng/ml) were excluded, the correlation between salivary morning CRP and serum CRP reached statistical significance (*r_s_*=0.24, *p*<0.05).

**Table 3:** Correlations between serum C-reactive protein (CRP), salivary CRP, and salivary CRP/total protein ratio calculated by Spearman’s (*r_s_*).

	Salivary CRP; morning	Salivary CRP; morning (% of total protein)	Salivary CRP; evening	Salivary CRP; evening (% of total protein)
Serum CRP	*r_s_* = 0.19	*r_s_* = 0.20	*r_s_* = 0.27**	*r_s_* = 0.20*
	*n* = 99	*n* = 99	*n* = 97	*n* = 97
Serum CRP (≤3 mg/mL)	*r_s_* = 0.19	*r_s_* = 0.20	*r_s_* = 0.28**	*r_s_* = 0.20*
	*n* = 80	*n* = 80	*n* = 79	*n* = 79
Serum CRP (>3 mg/mL)	*r_s_* = 0.25	*r_s_* = 0.14	*r_s_* = 0.54*	*r_s_* = 0.36
	*n* = 19	*n* = 19	*n* = 18	*n* = 18

Values are shown for all samples as well as for the low and high serum CRP levels (≤3 mg/ml, >3 mg/L).

*p < 0.05.

**p < 0.01.

### Associations Between Salivary/Serum C-Reactive Protein and Plasma IL-6

Detectable IL-6 values ranged from 1.77 to 22.93 pg/ml and median IQR for all samples was 0.56 (0.56–2.85). As excepted, participants with high serum CRP (>median) had significantly higher IL-6 levels (*p*=0.03) than those with lower serum CRP concentrations (≤median). For all available samples (*n*=97), IL-6 levels correlated with serum CRP (*r_s_*=0.41, *p*<0.01); however, when samples with IL-6 below detection limit were excluded the significance did not remain (**Table 4**). Using median split of daily mean salivary CRP values, we found no difference in IL-6 levels (*p*=0.30, *n*=98). These results remained essentially constant when testing separately for morning and evening saliva values (data not shown). In concordance, neither morning nor evening salivary CRP did correlate significantly with IL-6 levels (**Table 4**).

**Table 4:** Spearman (*r_s_*) correlations between serum C-reactive protein (CRP), salivary CRP, BMI and plasma IL-6.** **

	Serum CRP	Salivary CRP; morning	Salivary CRP; evening
BMI	*r_s_* = 0.43**	*r_s_* = 0.06	*r_s_* = 0.15
	*n* = 96	*n* = 99	*n* = 97
IL-6	*r_s_* = 0.41**	*r_s_* = −0.04	*r_s_* = 0.06
	*n* = 97	*n* = 98	*n* = 96
IL-6^#^	*r_s_* = 0.10	*r_s_* = 0.23	*r_s_* = 0.22
	*n* = 35	*n* = 35	*n* = 35

Subjects with undetectable IL-6 levels were given half the detection limit’s value. Additionally, correlations were calculated only for participants with detectable IL-6.

^#^IL-6 above detection limit.

**p < 0.01.

### Associations Between C-Reactive Protein, Body Mass Index and Tobacco Smoking

We found no significant difference in BMI levels between participants with low salivary and high salivary CRP (*p*=0.59, *n*=99). Higher BMI levels were found among participants with high serum CRP (>median), whereas those with low serum CRP concentrations (≤median) had lower BMI levels (*p*=0.01, *n*=96). CRP in serum correlated with BMI (*r_s_*=0.43, *p*<0.01), whereas morning and evening salivary CRP values showed no significant correlations with BMI (**Table 4**). Regarding tobacco smoking, non-smokers had significantly higher daily mean salivary CRP levels than smokers did (*p*=0.015, *n*=98; IQR=2.30 (1.11–7.74) and 1.13 (0.5–3.71) ng/ml, respectively). As compared to smokers, non-smokers showed borderline significant higher CRP using morning (*p*=0.08) and evening (*p*=0.05) saliva. In contrast, serum CRP levels were found to be independent of smoking (*p*=0.27).

### Specificity and Sensitivity of Salivary C-Reactive Protein to Identify Elevated Serum C-Reactive Protein

By using the 90^th^ percentile of serum CRP values among the 107 participants, a cut-off of 5.0 mg/L was achieved and used to define “systemic inflammation” based on laboratory findings. As demonstrated in **Table 5**, the specificity of both morning and evening saliva to detect individuals with biomarker-defined systemic inflammation was decent. However, the sensitivity was poor, particularly regarding morning saliva.

**Table 5:** Performance of salivary morning and evening C-reactive protein (CRP) to identify “systemic inflammation”, defined by the 90^th^ percentile of serum CRP values.

	Sensitivity	Specificity	Accuracy
Salivary CRP; morning (*n*=105)	0.10 (0.11–0.31)	0.89 (0.85–0.91)	0.82 (0.77–0.85)
Salivary CRP; evening (*n*=103)	0.22 (0.18–0.43)	0.91 (0.87–0.93)	0.85 (0.81–0.88)

Sensitivity, specificity and accuracy, including 95% confidence intervals (in parentheses) are given.

## Discussion

In the present study, we analyzed CRP and total protein in saliva from middle-aged individuals of the general population. A striking finding was the noticeable diurnal variation in salivary CRP and total protein with higher levels in mornings compared to evenings. Another observation was the considerable range of CRP in saliva and dilution factors had to be applied for some samples. These high levels of CRP were not be explained by visible blood contaminations, but might be due to acute local or systemic inflammatory processes. The observed similarity of CRP levels in saliva of men and women is in accordance with previous observations in serum (27) and saliva (23). Further, associations between aging and elevated CRP levels in serum have been reported in several studies (28) whereas such connection was not found in saliva (19, 21). In contrast, however, we found a dependency of age also in saliva as the 60–69 year old participants had significantly higher salivary CRP levels as compared to the 45–59 year old group.

The finding of diurnal variation in salivary CRP is in line with earlier studies, which suggested a similar pattern and, importantly, used comparable sampling times at awaking and bedtime (20, 23). It has been reported that serum levels of CRP are also affected by diurnal variations with a peak at 3pm and slightly higher levels in the morning than in the evening (27). In contrast, some smaller studies indicate that serum levels do not vary during the day (29, 30). We found a similar diurnal pattern for the salivary total protein concentrations, with a clear decrease toward the evening. Again, this is concordant to another study with similar sampling time points (23) whereas studies with later morning sampling hours and fewer participants reported stable, or even rising, total protein concentrations over the day (31, 32).

Adjustment for total protein concentrations affected the diurnal pattern of salivary CRP. As both variables decreased toward the evening, the ratio was not significantly different over the day when all samples were considered. However, the cut-off at 1.3 ng/ml of morning CRP, the ratio was decreasing toward the evening and contrariwise rising below this cut-off. As the low salivary CRP levels could be considered as baseline levels present in all healthy subjects, the differences in the low CRP subgroup could probably be regarded as a normal fluctuation. The low CRP subgroup contained mainly subjects with levels below the detection limit in the morning where a decrease toward the evening was not observed. It seems unlikely that the fall within the high CRP subgroup is only generated by higher dilution of saliva in the evening as there was still a decrease after correction for total protein. Furthermore, contrasting daily patterns for other salivary proteins such as α-amylase have been reported (33). Interestingly, the same authors showed that α-amylase also decreased strongly in the first 30 min after awaking and then started to rise over the day with the highest values in the evening.

To settle a standardized protocol of saliva sampling, the collection method is of utmost importance. Mechanical stimulation achieves higher flow rates than the passive drooling (34) and can be more comfortable for the donor. It seems that CRP and total protein levels do not differ between these two different collection methods or the use of salivettes (34, 35). It should be noticed that the sampling method used in this study is somewhat in between passive collection and mechanical stimulation as the sampling was performed using cotton swabs. Only in cases of too low flow rate, the participants were chewing on the swab and thus stimulating salivation. Taken together it seems beneficial to use salivettes, but not entirely necessary. Our results accentuate the need to standardize a sampling protocol and especially the sampling time point.

The CRP saliva-to-serum concentration ratio of the present study is in fairly agreement with that of a previous study, 1:1,084 versus 1:1,663 (19). Positive correlation between salivary and serum CRP have initially been reported in animal studies, such as in pigs (36). Since then several studies in healthy and diseased individuals investigated this association in humans with diverging results. One group reported no significant correlation between CRP in serum and saliva in healthy donors, although there was a trend toward it among a subgroup with high CRP levels (22). Opposed to them, other studies indicated moderate to strong correlations in healthy donors and in women exposed to intimate partner violence (19, 20), and further correlation was found in patients with ischemic heart disease (21). Our results present a low to moderate correlation between serum and salivary evening CRP, but stronger when stratifying for high serum CRP. Morning CRP did only show significant correlation when individuals with high salivary CRP levels (>10 ng/ml) were excluded. Very high salivary CRP morning levels could be due to such an acute phase reaction, already having started to decrease at the time of serum collection, and vice versa. Low levels of salivary CRP has been shown to predict low plasma levels more accurately than high salivary levels predicted high serum concentrations (20). Based on our data, we conclude that serum and salivary CRP levels do correlate but to a lower extent than previously reported. Only in subjects of the 60–69 year age interval, the statistical analyses suggested interchangeability between serum and saliva CRP.

Smoking, BMI and plasma IL-6 are known to be associated with systemic inflammation. Earlier, such correlations were reported also for salivary CRP and those variables (19). A study of women exposed to intimate partner violence could confirm the results for BMI, but not for smoking (21), contrasting our results without significant association between salivary CRP and BMI. The higher salivary CRP levels among non-smokers was unexpected and warrants further investigation. Nevertheless, anti-inflammatory properties of nicotine have been documented in obesity and nicotine-mediated modulation of hypothalamic-pituitary-adrenal axis activity was suggested as a potential mechanism (37, 38).

An essential issue when considering salivary CRP as a biomarker is to understand where it’s produced and/or the route of CRP entry to saliva – a question that has not been fully elucidated. Because of its high molecular weight and relative insolubility in lipids, it seems unlikely that CRP enters by diffusion or ultrafiltration through the tight junctions of the cells from the circulation (15, 16). The entry *via* the gingival crevicular fluid (GCF), a liquid found in the gingival sulcus between free gingival and the tooth is one possibility. CRP in GCF has been reported to be of systemic origin (39). The presence of CRP in GCF could be a result of systemic inflammation induced by periodontitis or disease elsewhere in the body. Salivary CRP levels may be elevated in patients with periodontal diseases (40), which emphasizes the need to consider oral health in following studies. Although the main origin of CRP is the liver, there have also been signs of CRP production by the salivary glands as indicated by elevated mRNA levels (41). The correlation of salivary CRP with detectable circulating IL-6, however, is another indication for derivation from the liver. It could be that by some unknown mechanism CRP is attracted to local oral sites of inflammation without affecting the systemic CRP concentrations.

The study has some limitations that should be mentioned. We acknowledge the lack of data on oral health status and salivation rate, which both might have biased the results and should be considered as limitations. Furthermore, sera were generally collected 3 to 4 days after the saliva used for CRP determination (*d*_3_). It cannot be excluded that this procedure could have affected the results. Finally, the definition of “systemic inflammation” was based on serum CRP levels only (no physical examination of the participants was performed). However, all individuals with symptoms of infection or severe disease had already been excluded (24).

To summarize, we observed evident diurnal variation of salivary CRP and total protein with higher levels at awaking time in the mornings compared to evenings. This highlights the need to standardize collecting times before introduction in clinical routine. The correlation between saliva and serum was low to moderate; stronger in the subgroup of serum CRP >3 mg/L, but interchangeability was suggested only among older participants. We found no clear impact of sex, but higher age and non-smoking were associated with increased levels of salivary CRP. The sensitivity of salivary CRP to detect individuals with elevated serum CRP was not impressive. In contrast to serum CRP, salivary CRP did not correlate with BMI or plasma IL-6. Further investigations are needed to clarify the relevance and suitability of CRP (and possibly other pentraxins) as salivary biomarkers before reliable introduction in clinical routine is possible.

## Data Availability Statement

The raw data supporting the conclusions of this article will be made available by the authors, without undue reservation.

## Ethics Statement

The LSH study protocol was approved by the regional ethics review board in Linköping (No. 02–324). The patients/participants provided their written informed consent to participate in this study.

## Author Contributions

All authors were involved in drafting the article or revising it critically for important intellectual content, and all authors approved the final version to be published. JW and CS had full access to all of the data in the study and takes responsibility for the integrity of the data and the accuracy of the data analysis. JW, SL, MK, PG, and CS conceptualized and designed the study. SL, MK, and PG acquired the data. JW, SL, FC, MK, PG, and CS analyzed and interpreted the data. All authors contributed to the article and approved the submitted version.

## Funding

This work was supported by grants from the Swedish Rheumatism Association (R-844801), the Region Östergötland (ALF Grants) (LiO-932055), the Swedish Society of Medicine (SLS-590231), the King Gustaf V’s 80-year Anniversary foundation (FAI-2018-0504) and the King Gustaf V and Queen Victoria’s Freemasons’ foundation.

## Conflict of Interest

The authors declare that the research was conducted in the absence of any commercial or financial relationships that could be construed as a potential conflict of interest.
